# The role of specific isoforms of Ca_V_2 and the common C-terminal of Ca_V_2 in calcium channel function in sensory neurons of Aplysia

**DOI:** 10.1038/s41598-023-47573-z

**Published:** 2023-11-18

**Authors:** Tyler W. Dunn, Xiaotang Fan, Jiwon Lee, Petranea Smith, Rushali Gandhi, Wayne S. Sossin

**Affiliations:** Department of Neurology and Neurosurgery, Montreal Neurological Institute, McGill University, Montreal, QC H3A 2B4 Canada

**Keywords:** Biochemistry, Molecular biology, Neuroscience

## Abstract

The presynaptic release apparatus can be specialized to enable specific synaptic functions. Habituation is the diminishing of a physiological response to a frequently repeated stimulus and in *Aplysia*, habituation to touch is mediated by a decrease in transmitter release from the sensory neurons that respond to touch even after modest rates of action potential firing. This synaptic depression is not common among *Aplysia* synaptic connections suggesting the presence of a release apparatus specialized for this depression. We found that specific splice forms of ApCa_V_2, the calcium channel required for transmitter release, are preferentially used in sensory neurons, consistent with a specialized release apparatus. However, we were not able to find a specific ApCa_V_2 splice uniquely required for synaptic depression. The C-terminus of ApCa_V_2 alpha1 subunit retains conserved binding to Aplysia rab-3 interacting molecule (ApRIM) and ApRIM-binding protein (ApRBP) and the C-terminus is required for full synaptic expression of ApCa_V_2. We also identified a splice form of ApRIM that did not interact with the ApCav2 alpha 1 subunit, but it was not preferentially used in sensory neurons.

## Introduction

Synapses are specialized for their function. From the presynaptic perspective, synapses display great diversity in their probability of release, presynaptic plasticity, reliability, and virtually any parameter one can choose that characterizes presynaptic properties^1–3^. These specializations often require distinct presynaptic molecules present at synapses, either distinct proteins or more commonly distinct isoforms of the constituents of the active zone^4^. The presynaptic release apparatus, often called the active zone, is made up of a number of scaffolds and proteins implicated in priming of synaptic vesicles and the coupling of calcium influx to the calcium-dependent closing of the SNARE complex that causes synaptic vesicle release^5^. These proteins include the calcium channel itself, proteins implicated in regulating the priming of synaptic vesicles such as Unc13, Unc18, and a large number of proteins involved in localizing calcium channels to synaptic vesicles, such as ELKS/CAST/Bruchpilot, RIM and both RIM-binding proteins and RIM-related proteins (Piccollo/Fife), transmembrane proteins (Liprin, Mint, Neurexin) and the calcium sensor synaptotagmin^5,6^. In some cases, specialized aspects of transmitter release have been shown to be regulated by the distinct type of calcium channel^7^, or variants of the synaptic vesicle priming factor Unc-13^8^, or the relative levels of RIM-like proteins^9^, or distinct forms of synaptotagmin^10^. Thus, presynaptic terminals specialized for a unique function would be expected to have a distinct complement of active zone proteins.

One of the first types of memory that was linked to synaptic plasticity was the habituation of the gill withdrawal reflex in *Aplysia*. It was shown that repetitive touches to the siphon led to a reduced withdrawal of the gill and that this was mediated by a decrease in transmitter release by the siphon sensory neurons^11,12^. This reduction in transmitter release is a general feature of the sensory neurons of *Aplysia* and is also observed when the sensory neurons for the tail and body are used to make isolated sensory to motor neuron cultures^13,14^. This reduction in transmitter release with low frequency firing observed in isolated cultures was termed homosynaptic depression (HSD), as it does not involve the release of modulatory transmitters from other neurons. Considerable research has focused on the mechanism underlying this surprising decrease in transmitter release. The synaptic depression occurs through a reduction in quantal content^12^ and is independent of post-synaptic currents as blocking post-synaptic glutamate receptors does not affect the rate of depression^15^. While several modeling studies suggested HSD was due to the presynaptic ‘silencing’ of a subset of synapses^16,17^, direct measurement of release sites using excitatory postsynaptic calcium transients do not support this idea^18,19^. There is no change in the amplitude of the action potential-associated calcium transient in the presynaptic cell with synaptic depression, though an increase has been reported with the application of 5HT^20,21^. A reduction in the readily releasable pool of synaptic vesicles has been reported, but not sufficient to account for the full extent of depression observed, indicating that the reduction in transmitter release is partially through a reduction in the readily releasable pool of synaptic vesicles and partially through a reduction in calcium-secretion coupling efficiency^22^. This may be due to differences in the molecules at the synapses that undergo depression, and thus understanding the sub-synaptic molecular interactions at this synapse should provide further insights into the mechanisms by which heterogeneity in synaptic transmission is achieved.

Most synapses in *Aplysia* do not exhibit homosynaptic depression^23–32^. Thus, there is likely to be a specialized set of active zone proteins present (or absent) at the sensory neuron synapse to support the specialized depression that underlies behavioral habituation. In particular, we have previously noted six sites of alternative splicing in the alpha subunit of ApCa_V_2 (ApCa_V_2a1)^33^, consistent with the possibility that specific forms of the channel may support specialized release as is seen in other synapses^7^. Moreover, a portion of homosynaptic synaptic depression occurs through a reduction in calcium-secretion coupling that could reflect changes in the proximity of the calcium channel to the synaptic vesicle^22^. Here, we determine that there is enrichment for specific splice forms of ApCa_V_2a1 and for some other synaptic scaffold proteins, such as the exclusion of the RIM-related Fife protein in sensory neuron compared to the rest of the nervous system. We focused on the C-terminal region of ApCa_V_2a1 and confirmed a conserved binding site for RIM and RIM-binding protein (RBP) in this region of the channel. We tested splice forms of ApCa_V_2a1 for changes in homosynaptic depression and examined homosynaptic depression when a large part of the C-terminal region of ApCa_V_2a1is removed, but due to the requirement for the C-terminal for synaptic expression, a role of the C-terminal in depression could not be fully examined.

## Results

### Intron/Exon boundaries are highly conserved, but not sites of alternative splicing

There has recently been improved genome and transcriptome resources available for *Aplysia*^34^. Using these resources, we identified 49 coding region exons and 5 untranslated region exons encoding ApCa_V_2a1 and confirmed the presence of all eight previously identified sites of alternatively spicing^33^ in the genome (Supplementary Table 1). These eight sites involve six sites where either an exon is present or not, one site where one of two alternative exons is used, and one site where either no exon or two alternative exons is used. Together with the four alternative start sites based on their distinct 5’untranslated region exons, this suggests over 700 (2^7^ × 3 × 4) possible ApCa_V_2a1 isoforms from this gene. We compared the exon/intron organization to other species and found that while there is strong conservation of intron/exon boundaries in the pore forming region of ApCa_V_2a1, many present since before the diversion of Ca_V_2 from other calcium channels^35,36^, there is less conservation of intron/exon boundaries in the C-terminal and in the linker between the II and III ion channel repeats and these are the sites for much of the alternative splicing and thus these splicing events are only seen in other Molluscs^33^ (Supplementary Table 1). One exception is a splice in ion channel repeat III (Exon 27, Supplementary Table 1) that appears to be conserved throughout Bilateria^37^.

### Sensory neurons are enriched for specific isoforms of ApCa_V_2a1

**Figure 1:**
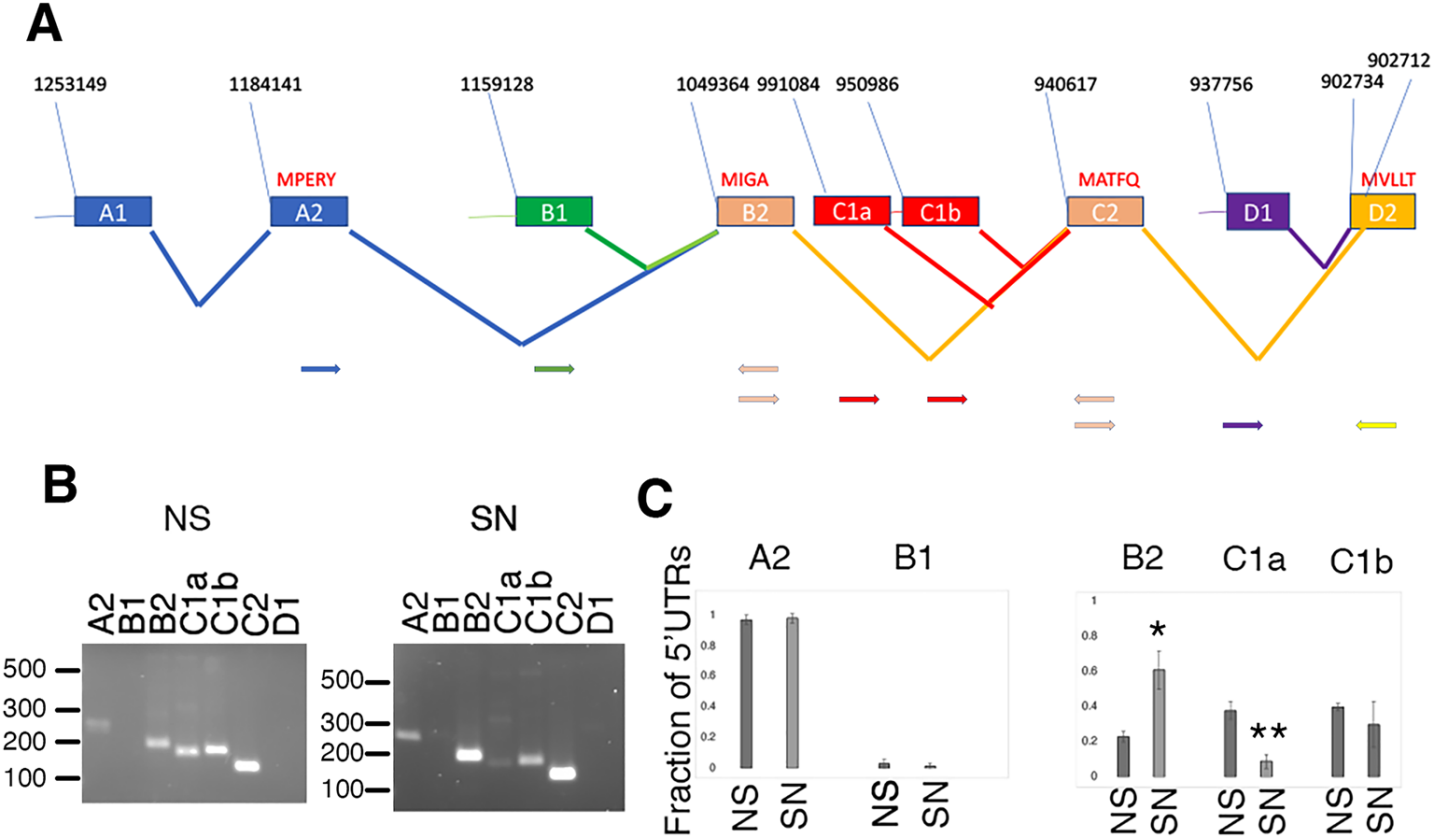
Transcriptional start sites of ApCa_V_2a1. (**A**) Genomic organization of transcriptional start sites for Aplysia Ca_V_2a1. Sequence numbers come from the Aplysia genome blast site (HiC scaffold: http://aplysiatools.org:8080/). Sequences are all found on scaffold HiC_scaffold_12 Presumed initiating methionines and first amino acids are pictured in red in the first coding exon for that start site. Pictured below are the approximate locations of the primer sets used to determine the proportion of transcriptional starts. A2 and B1 have a common 3’primer in B2; B2 C1a and C1b have a common 3’ primer in C2 and C2 and D1 have a common 3’ primer in D2 (see Supplemental Table 2 and Methods). (**B**) Agarose gels with PCR products from amplifications to test for the proportions of start sites for one cDNA library for sensory neurons (SN) and total nervous system (NS). Uncropped gels are shown in Supplemental Fig. 1. (**C**) Quantification of three separate amplifications from three independently generated cDNA libraries from sensory neurons (SN) and total nervous system (NS). Fraction of 5’UTRs. Statistical comparisons are two-tailed t-tests between SN and NS for each splice site, *p < 0.05, **p < 0.01 after Bonferroni corrections for multiple tests (5). If not shown test was not significant p > 0.05. Error bars are S.E.M.

To determine if distinct forms of ApCa_V_2a1 are enriched in the sensory neurons, we first derived primers to differentiate the use of alternative start sites (Supplementary Table 2). Since each start site has a unique 5’UTR, we derived a common reverse primer and unique forward primer located in the 5’UTR of each identified transcriptional start site (Fig. 1A). We then compared the amount of PCR product from the unique forward primer in the 5’UTR and the common reverse primer with a forward primer in the upstream exon and the common reverse primer. This compares the relative amount of a transcript starting in that start with all the longer start sites that contain the upstream exon. We did not detect use of the shortest start site, that would start the channel right before the first ion channel repeat (Fig. 1B). In the total nervous system, over 50% of the transcripts use the start site previously described^33^. This percentage is significantly less in the sensory neurons (Fig. 1B quantified in Fig. 1C) due to the low use of the transcriptional start site before exon C1a. We detected only small amounts of the second start site (Fig. 1B) so most remaining transcripts use the first start site, which is thus more prevalent in sensory neurons than the rest of the nervous system (Fig. 1C).

**Figure 2:**
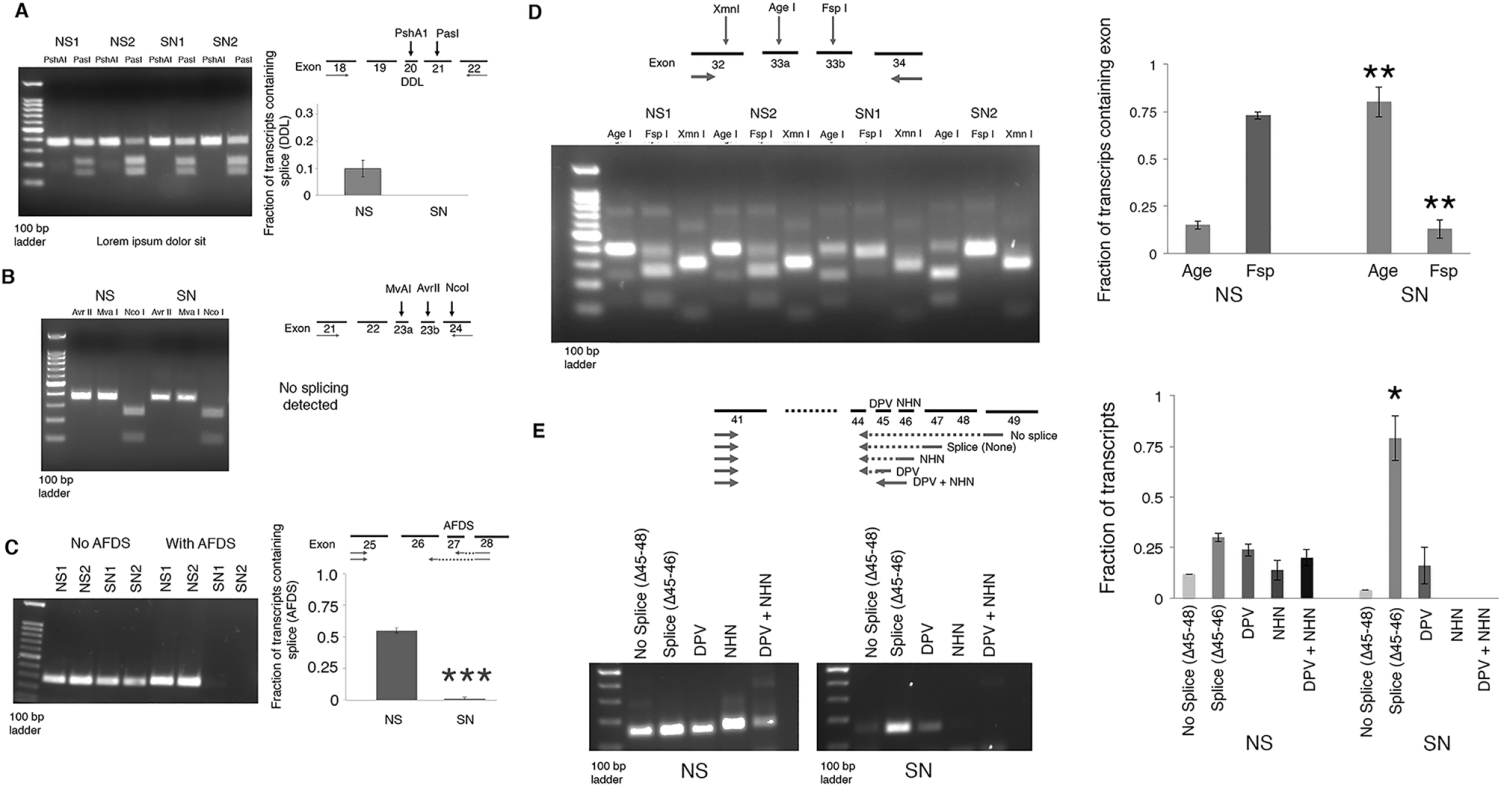
Examination of preferential splice site usage in sensory neurons for ApCa_V_2a1. Each part shows the strategy used to determine the splicing status, an example agarose gel showing the results (left) and quantification of the fraction spliced in the nervous system (NS) or sensory neurons (SN) (right). (**A**) Alternative inclusion of exon 20 including the three amino acids aspartate, leucine, leucine (DLL). (**B**) Alternate inclusion of either exon 23a, 23b, or no exon. (**C**) Alternate inclusion of exon 27 including alanine, phenylalanine, aspartic acid, serine (AFDS), (**D**) use of either exon 33a or exon 33b. (**E**) Alternate inclusion of a subset of possible uses of exons 45–48, No splice (No inclusion of exons 45–48); Splice only (includes exon 47, but not 45 or 46), includes exon 46, but not exon 45 starting with asparagine, histidine, asparagine (NHN), includes exon 45 starting with aspartic acid, proline, valine (DPV), includes exons 45 and 46 (DPV + NHN); See Supplemental Table 3. Different numbers, i.e. NS1 and NS2, refers to different cDNA libraries. Value in graphs represent average with S.E.M. N = 3–5 separate cDNA libraries. Statistical comparisons are two-tailed t-tests between SN and NS for each splice site *p < 0.05, **p < 0.01, ***p < 0.001. Bonferroni corrections for multiple tests was used for E (5). If not shown test was not significant p > 0.05. Uncropped agarose gels are shown in Supplemental Fig. 1.

We then designed primers to assess the inclusion of differentially spliced exons allowing comparison in the percentage splicing between overall nervous system and sensory neurons. In most cases, we took advantage of differences in restriction enzyme sites in the distinct splice isoforms and then compared the percentage of the PCR product cut with the different enzymes. For the use of exon 27 and exons 45–48, we used a common forward primer coupled to distinct reverse primers that distinguished between the splice sites but amplified products of approximately the same size (Supplemental Tables 2, 3). While these strategies may not faithfully represent the absolute percentage of splicing, they should be sufficient to determine differences between sensory neurons and nervous system. We only detected a small amount of inclusion of exon 20 containing the three amino acids DDL, although this is stronger in the nervous system than in sensory neurons (Fig. 2A). We did not detect either exon 23a or 23b in sensory neurons or the total nervous system (Fig. 2B). Both these alternative splices are in the linker region between the second and third repeat of the channel. In contrast, while the nervous system showed extensive use of exon 27 (inclusion of the amino acids AFDS in an extracellular loop between TM2 and TM3 in ion channel repeat 3), the sensory neurons showed no detectable inclusion of this exon (Fig. 2C). In the fourth ion channel repeat, there are two alternative exons coding for the loop between TM1 and TM2 and the second TM domain. The total nervous system and sensory neurons favor distinct exons for this region (Fig. 2D). Finally, there are four exons in a row in the proximal C-terminal of the channel following the IQ domain that are all alternatively used (Supplemental Tables 2, 3). The sensory neurons use less of exon 46 and less of a form that skips all the exons compared to the nervous system (Fig. 2E). Thus, at most of the splice sites we were able to detect using these experiments, sensory neurons had a different pattern of splicing than the overall nervous system suggesting a specialized ApCa_V_2a1 isoform in sensory neurons.

**Figure 3:**
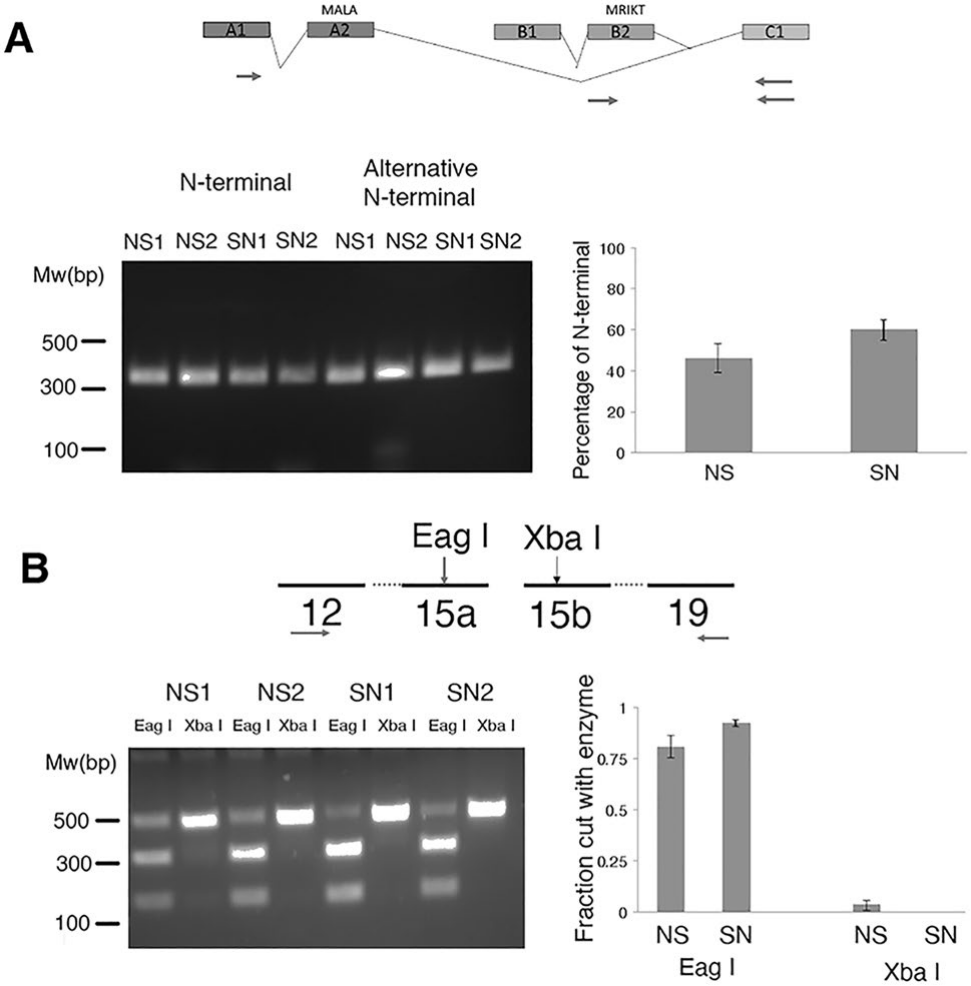
Examination of preferential splice site usage in sensory neurons for Aplysia Ca_V_2 beta and alpha2-delta subunits. Each part shows the strategy used to determine the splicing status, an example agarose gel showing the results (left) and quantification of the fraction spliced in the nervous system (NS) or sensory neurons (SN) (right). (**A**) Determination of percentage of transcripts starting at splice site A or B. (**B**) Use of either exon 15a or exon 15b. Different numbers, i.e. NS1 and NS2, refers to different cDNA libraries. Value in graphs represent average with standard error of the mean. N = 3–5 separate cDNA libraries. Statistical comparisons are two-tailed t-tests between SN and NS for each splice site. No test was significant p > 0.05. Uncropped agarose gels are shown in Supplemental Fig. 1.

There are two additional subunits of Ca_V_2 channels, a beta subunit and an alpha-2/delta subunit. We had previously found an alternative splice site in alpha-2/delta and an alternative start site in the beta subunit that is highly conserved over evolution^33,38^. There is no differential use of this start site for the beta subunit in sensory neurons, nor preference for a splice site in alpha-2/delta subunit (Fig. 3).

### The C-terminal of the channel is required for synaptic expression of ApCa_V_2a1

**Figure 4:**
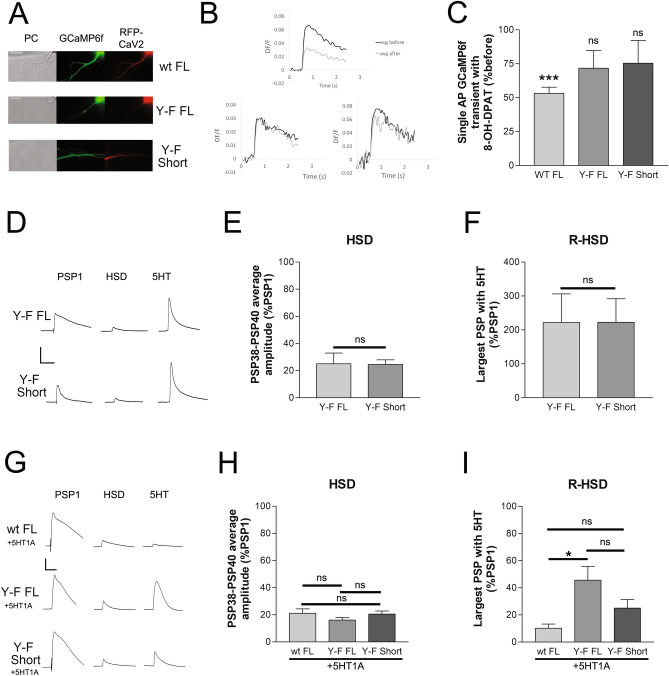
Characterization of ApCa_V_2a1 short. The participation of a C-terminal truncated RFP-ApCa_V_2a1 short isoform to the single isolated sensory neuron calcium transient is similar to full-length RFP-ApCa_V_2a1 but does not effectively contribute to the synaptic calcium current that triggers transmitter release. (**A**) Representative images of sensory neuron processes in phase contrast (PC) expressing 5HT1A receptor, GCaMP6f and either wildtype full-length RFP-ApCa_V_2a1 (wt full), RFP-ApCa_V_2a1 Y-F full-length (Y-F full), or RFP-ApCa_V_2a1 Y-F short C-terminus (Y-F short). Scale bar 20 μm. (**B**) Representative single action potential GCaMP6f transients before and after application of the 5HT1A agonist 8-OH-DPAT. Mean initial transient peak amplitudes were 0.051 ± 0.008, 0.041 ± 0.007, 0.067 ± 0.012 DF/F for RFP-ApCa_V_2a1 wt full-length, RFP-ApCa_V_2a1 Y-F full-length, and RFP-ApCa_V_2a1 Y-F short respectively, and not significantly different from one another (P > 0.05, comparing groups with t-tests). (**C**) Inhibition of the GCaMP6f calcium transient with 8-OH-DPAT mediated 5HT1A activation in sensory neurons expressing either RFP-ApCa_V_2a1 wt full-length, or RFP-ApCa_V_2a1 Y-F full-length, or RFP-ApCa_V_2a1 Y-F short. Inhibition of the GCaMP6f transient with 5HT1A activation was significant in the sensory neurons expressing RFP-ApCa_V_2a1 wt full-length comparing peak GCaMP6f transient before to after application of 8-OH-DPAT (P = 0.0011), but not in sensory neurons expressing RFP-ApCa_V_2a1 Y-F full-length (P = 0.1165) or RFP-ApCa_V_2a1 Y-F short (P = 0.4386), n = 6,6,6. (**D**) Representative traces of the initial PSP (PSP1), PSP 40, and a PSP following 5HT application at sensory neuron to motor neuron synapses expressing either RFP-ApCa_V_2a1 Y-F full-length or RFP-ApCa_V_2a1 Y-F short in the sensory neuron. Initial PSP amplitude (12.3 ± 3.4 and 10.8 ± 2.7, respectively), PSP amplitude following homosynaptic depression (average PSP amplitude of the last three PSPs before 5HT) (**E**), and the recovery from HSD with 5HT (largest PSP following addition of 5HT) (**F**), was similar in both groups compared with t-tests (P > 0.05 for all three comparisons). (**G**) Representative traces of PSPs from sensory to motor neuron synaptic pairs expressing either RFP-ApCa_V_2a1 wt full-length + GFP-5HT1A or RFP-ApCa_V_2a1 Y-F full-length + GFP-5HT1A or RFP-ApCa_V_2a1 Y-F short + GFP-5HT1A of this first PSP (PSP1), a depressed PSP following HSD (HSD), and the largest PSP of 15 evoked following 5HT application (5HT). Initial PSP amplitude was not significantly different between the three groups at 42.5 ± 11.4, 16.2 ± 5.1, 36.7 ± 6.7, (F = 3.33) P > 0.05 determined with a one-way ANOVA. H) PSP amplitude following homosynaptic depression was also similar in all groups compared with a one-way ANOVA, P > 0.05 (F = 1.38). I) Application of 5HT at the depressed synapses led to significant facilitation of PSP amplitude (reversal of depression) in the group expressing RFP-ApCa_V_2a1 Y-F full-length (P < 0.05) when compared to expression of RPF-ApCa_V_2a1 wt full-length with a one-way ANOVA, (F = 5.29). Expression of the RPF-ApCa_V_2a1Y-F Short did not significantly reduce heterosynaptic inhibition with 5HT application like the full-length isoform, compared to expression RPF-ApCa_V_2a1 wt full-length (P > 0.05).

There is evidence that the C-terminal of Ca_V_2a1 is critical for surface expression, localization of the protein to synapses and for interactions important for priming and regulation of transmitter release^39^. To determine the role of the C-terminal of ApCa_V_2a1 in sensory neurons we generated a truncated channel that retained only the EF hand and IQ domains of the C-terminal (RFP-ApCa_V_2a1 short). We first tested for surface expression of the short channel in isolated sensory neurons in culture. To test for surface expression, we take advantage of our previous observation that expression of an exogenous channel can replace the surface expression of the endogenous channel. Heterosynaptic inhibition of the calcium current in *Aplysia* is mediated by Src phosphorylation of a conserved tyrosine in the EF hand of the channel^33,40^. Thus, replacement of the endogenous channel with the channel where the tyrosine phosphorylated by Src in the EF hand is converted to a non-phosphorylatable phenylalanine (RFP-ApCa_V_2a1 Y-F) leads to a reduction in heterosynaptic inhibition^33^. Only about half of the sensory neurons in cultures have receptors for dopamine and FMRFamide that allow examination of heterosynaptic inhibition^40^, so we also co-express the *Aplysia* 5HT1a receptor, and induce inhibition with an agonist of this receptor as we have done in previous experiments to ensure heterosynaptic inhibition is enabled^33^. Finally, to measure inhibition of the calcium current, we examined action potential-associated calcium transients with expression of the encoded calcium indicator GCaMP6f^18^. Sensory neurons were injected with expression vectors containing GCaMP6f, 5HT1A, and an RFP-tagged ApCa_V_2a1 then 48 h later, single action potential GCaMP6f transients were measured before and after addition of the 5HT1A agonist, 8-OHDPAT. There was significant inhibition of the GCaMP6f transients with expression and activation of 5HT1A when co-expressed with a ApCa_V_2a1 containing the wildtype phosphorylatable EF-hand Y residue (RFP-ApCa_V_2a1 wt full), however, co-expression of either full-length ApCa_V_2a1 with the Y-F mutation (RFP-ApCa_V_2a1 Y-F full) or the short isoform with the Y-F point mutation (RFP-ApCa_V_2a1 Y-F short) produced similar resistance to this 5HT1A-mediated inhibition (Fig. 4A–C). This suggests that the truncated channel is expressed on the plasma membrane and could replace the endogenous channel to a similar extent as the full-length channel. Expression of either the ApCa_V_2a1 full-length (RFP-ApCa_V_2a1 Y-F full) or short C-terminus (RFP-ApCa_V_2a1 Y-F short) in sensory neurons paired with postsynaptic motor neurons in culture exhibited homosynaptic depression (HSD) to a similar extent with forty stimuli at low frequency and reversal of HSD with application of 5HT (Fig. 4D–F), indicating normal plasticity with expression of the truncated channel. This would indicate that the C-terminal was not required for depression or the reversal of depression, but while the truncated channel can replace endogenous channels in the plasma membrane of isolated sensory neurons, it is unclear if the truncated channels replace Ca_V_2 channels expressed at the synapse. Thus, it was not clear if the lack of an effect on HSD and reversal of HSD with expression of the short channel is due to lack of a contribution of the truncated channel to the synaptic calcium transient or a lack of a role of the C-terminus of ApCa_V_2a1 in synaptic depression. To determine if the ApCa_V_2 short channel is participating in the synaptic calcium transient, the GFP-5HT1A receptor was co-expressed with either RFP-ApCa_V_2a1 subunits wt full, Y-F full, or Y-F short. Heterosynaptic inhibition is dominant to the enhancement of synaptic transmission that occurs with 5HT at depressed synapses (recovery from HSD), such that expression of 5HT1A and subsequent activation with 5HT will instead lead to further inhibition at depressed synapses through inhibition of the Ca_V_2 current^33,40,41^. RFP-ApCa_V_2a1 Y-F full-length was more effective than the RFP-ApCa_V_2a1 Y-F short C-terminus at relieving heterosynaptic depression induced by activation of co-expressed 5HT1A apparent with expression of the wt channel (RFP-ApCa_V_2a1 wt full-length)(Fig. 4C), indicating significantly less contribution of the truncated channel to the synaptic calcium transient (Fig. 4G–I). This suggests that the truncated channel is less effective at contributing the synaptic Ca_V_2 current as compared to the overall Ca_V_2 current, consistent with less synaptic localization of a Ca_V_2 channel when most of the C-terminal is absent. Thus, it remains unclear whether the C-terminus of the channel is important for the mechanisms underlying synaptic depression as expression of the short form does not effectively replace the endogenous channel at the synapse.

### Alternative splicing at exons 33 or 45–48 does not explain synaptic depression

**Figure 5:**
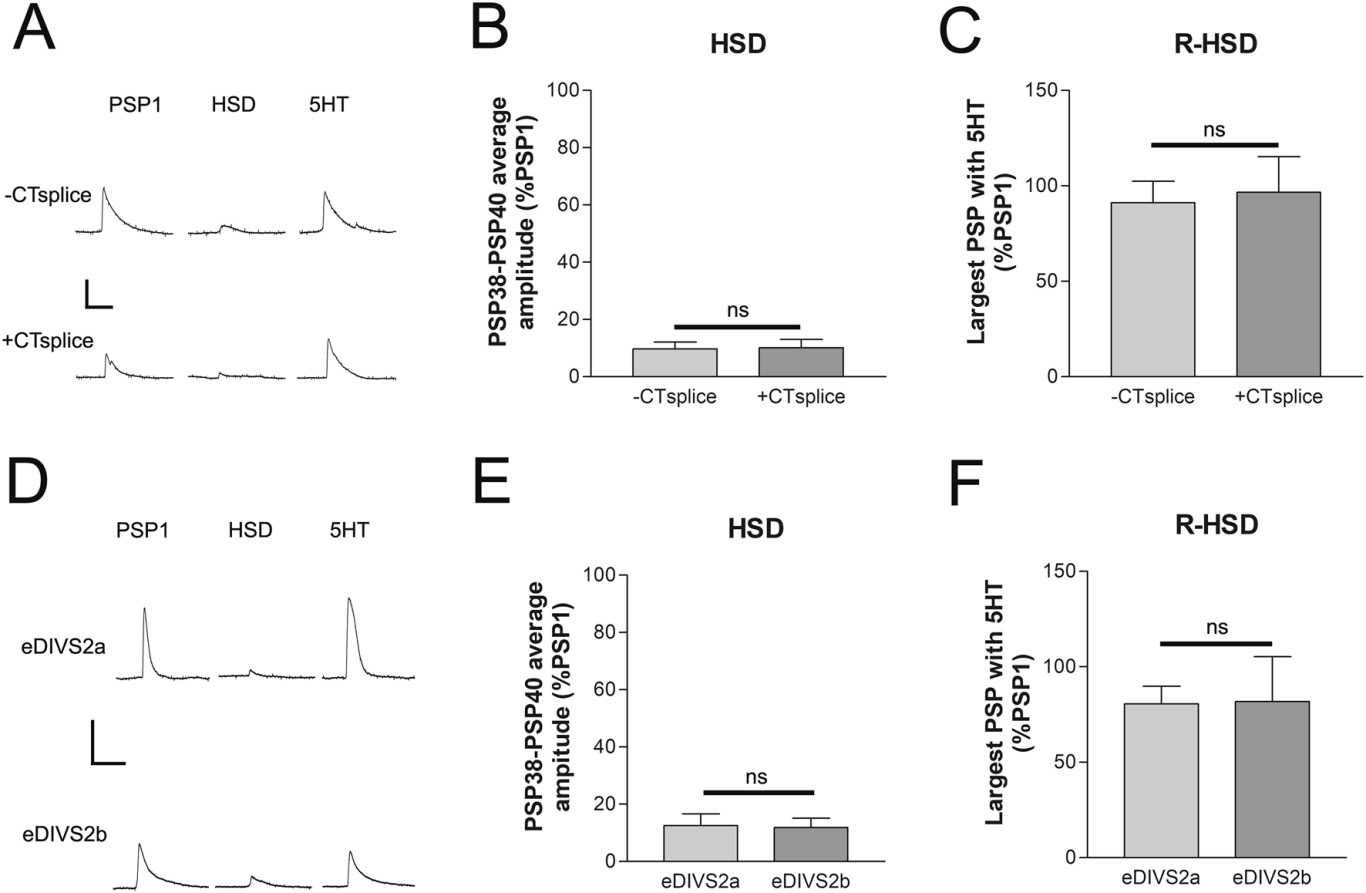
ApCa_V_2a1 splice variants in the C-terminus and in domain III have normal HSD and recovery from HSD with 5HT. (**A**) Representative traces of PSPs evoked at synapses where the sensory neuron was expressing either RFP-ApCa_V_2a1 without the C-terminal splice (CTsplice) or with the splice (+ CTsplice). The average initial PSP amplitudes of 11.2 ± 2.3 and 18.32 ± 6.8 respectively, were significantly different between the two groups (P = 0.038) when compared with a t-test, however, (**B**) the amount of homosynaptic depression measured between the two groups and the (**C**) amount of facilitation with 5HT following depression was similar for both -CTsplice and + CTsplice isoforms (P > 0.05, compared with t-tests, n = 4,5). (**D**) Representative traces of PSP1, a depressed PSP, and a PSP with 5HT following depression with expression of either RFP-ApCa_V_2a1 eDIVS2a or RFP-ApCa_V_2a1 eDIVS2b in the sensory neuron. Scale bars are 15 mV/100 ms. Initial PSP amplitude was similar in both groups at 14.6 ± 2.8 and 13.7 ± 2.3 mV, P > 0.05 compared with t-test. (**E**) Homosynaptic depression measured as the average of PSP37-39 was similar in both groups as was (**F**) the recovery from HSD measured as the largest PSP with addition of 10 μM 5HT, P > 0.05 compared with t-tests (n = 6,6).

The decreased presence of a ApCa_V_2a1 isoform lacking four exons (Exons 45–48) in the *Aplysia* sensory neurons (Fig. 2) suggested the possibility that expression of this longer isoform may participate in synaptic depression. To determine if these four exons alter synaptic depression we directly compared the amount of depression and reversal of depression in sensory neurons expressing a construct with all four exons expressed and one with none of the exons expressed. After expressing ApCa_V_2a1 for 48 h, we have previously demonstrated that the majority of channels present are the newly expressed channel^33^. However, we detected no difference in the rate or amount of synaptic depression when the expressed calcium channel contained or did not contain these exons (Fig. 5A–C). Given the non-conserved nature of these exons (Supplemental Table 1), it is unlikely that they are required for synaptic localization.

We also tested whether preferential expression of exon 33a or b (the two alternative exons for TM domain 2 in ion channel repeat 4) affected depression since this changes the ion channel itself. Similar to the C-terminal splice we did not detect any differences in depression or the reversal of depression based on whether the expressed channel included 33a or 33b (Fig. 5D–F).

### Binding interactions of RIM and RBP with Ca_V_2 are conserved in Aplysia

**Figure 6:**
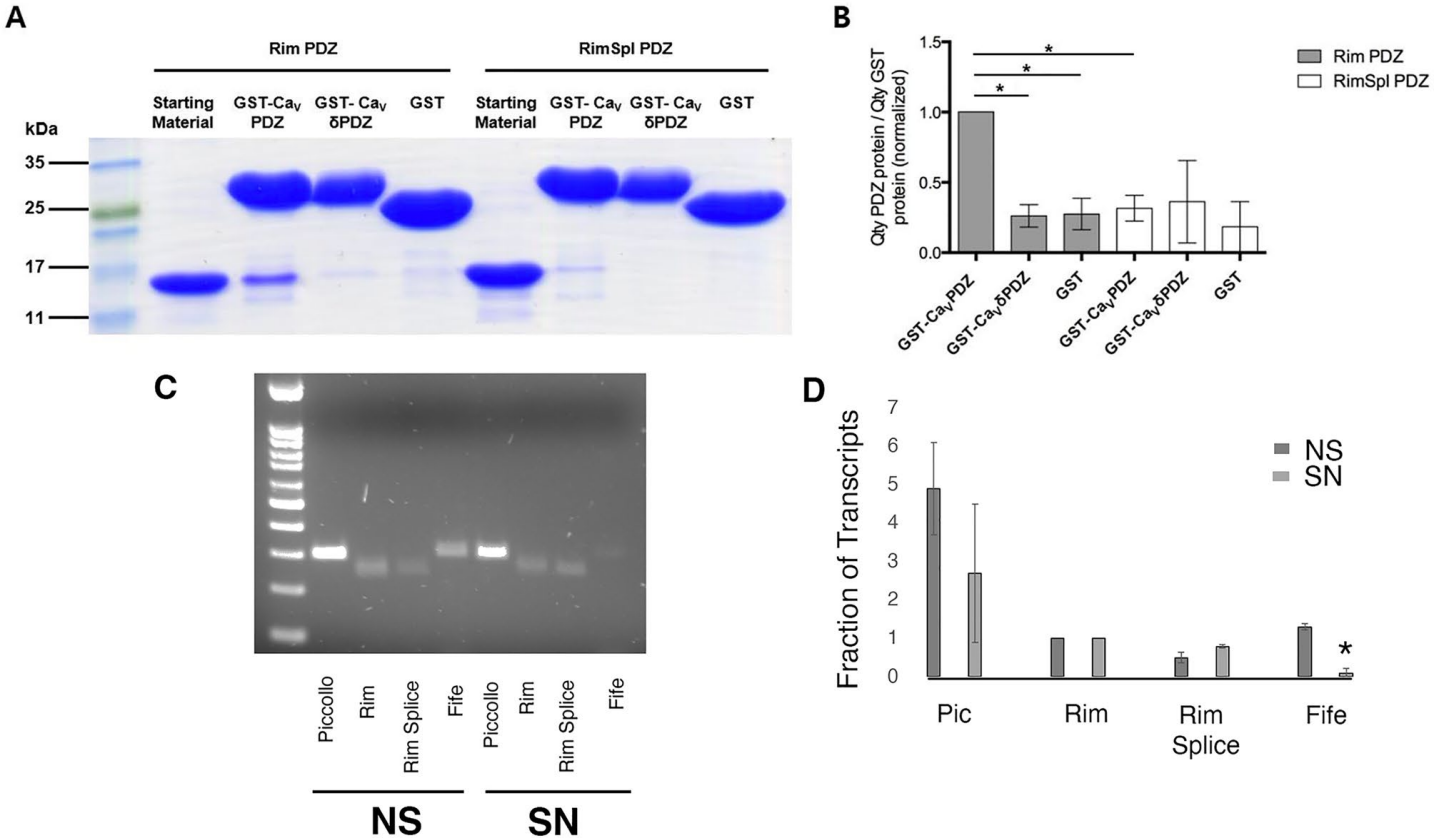
ApRIM binds the C-terminus of ApCa_V_2 alpha 1. (**A**) Coomassie stained gel of pull down of His-tagged PDZ domain of RIM or His-tagged PDZ-splice (See Supplemental Table 5 for sequence) with GST, GST-ApCa_V_2a1 PDZ (GST-CaV PDZ, C-terminal last 15 amino acids; Supplemental Table 5) or Gst-ApCaV2a1 ∆PDZ (GST-CaV δPDZ, C-terminal 15 amino acids with the last 4 amino acids (aspartic acid, aspartic acid, tyrptophan, cysteine (DDWC) removed). (**B**) Quantification of 5 experiments similar to (**A**) normalized to binding of ApCa_V_2a1 PDZ. Error bars are SEM. (One sample, one tailed Paired t-test comparing all groups to ApCa_V_2a1 PDZ with Bonferroni correction for multiple tests, *, p < 0.05). (**C**) Agarose gel example for Qualitative PCR examination for proportion of RIM proteins in Sensory neurons (SN) and nervous system (NS). Uncropped agarose gels are shown in Supplemental Fig. 1. (**D**) Quantification of PCR; the levels of products were normalized in each gel to the level of RIM from that cDNA library. Results are from three independent cDNA libraries, Error bars are SEM. Statistical comparisons are two-tailed t-tests between SN and NS for each RIM isoform *, p < 0.05 with Bonferroni corrections for multiple tests (4). If not shown test was not significant p > 0.05.

Since the C-terminal is important for synaptic expression of ApCa_V_2a1, we determined if known C-terminal interactions important for this were conserved. The last three amino acids of the C-terminal of Ca_V_2a1 (DWC) are conserved from Cnidarians to humans^35^ and this putative PDZ ligand has been shown to directly bind RIM in mammals^42^ and RIM has been shown to be important for Ca_V_2 localization to synapses in both *Drosophila*^43^ and *C. elegans*^44^. We have recently examined the RIM family over evolution and *Aplysia* has three RIM family members, a RIM ortholog, a Fife ortholog and a Piccolo ortholog^34^. We also found alternative exon usage in the PDZ domain of ApRIM that adds five amino acids preceding beta sheet B (Supplemental Table 4), a region implicated in ligand binding in RIM PDZ domains^35,45^. Using a GST fusion protein containing the last 40 amino acids either with or without the last three conserved amino acids “DWC”), we found that the PDZ domain of RIM, but not the PDZ domain containing the splice insert bound to this domain (Fig. 6A, quantified in Fig. 6B). Both these forms are expressed in sensory neurons at approximately the same level as in the rest of the nervous system (Fig. 6C, quantified in Fig. 6D). While the *Aplysia* ortholog of Piccolo is also expressed in both the nervous system and sensory neurons, the *Aplysia* Fife ortholog is expressed at lower amounts in sensory neurons (Fig. 6C. quantified in Fig. 6D).

**Figure 7:**
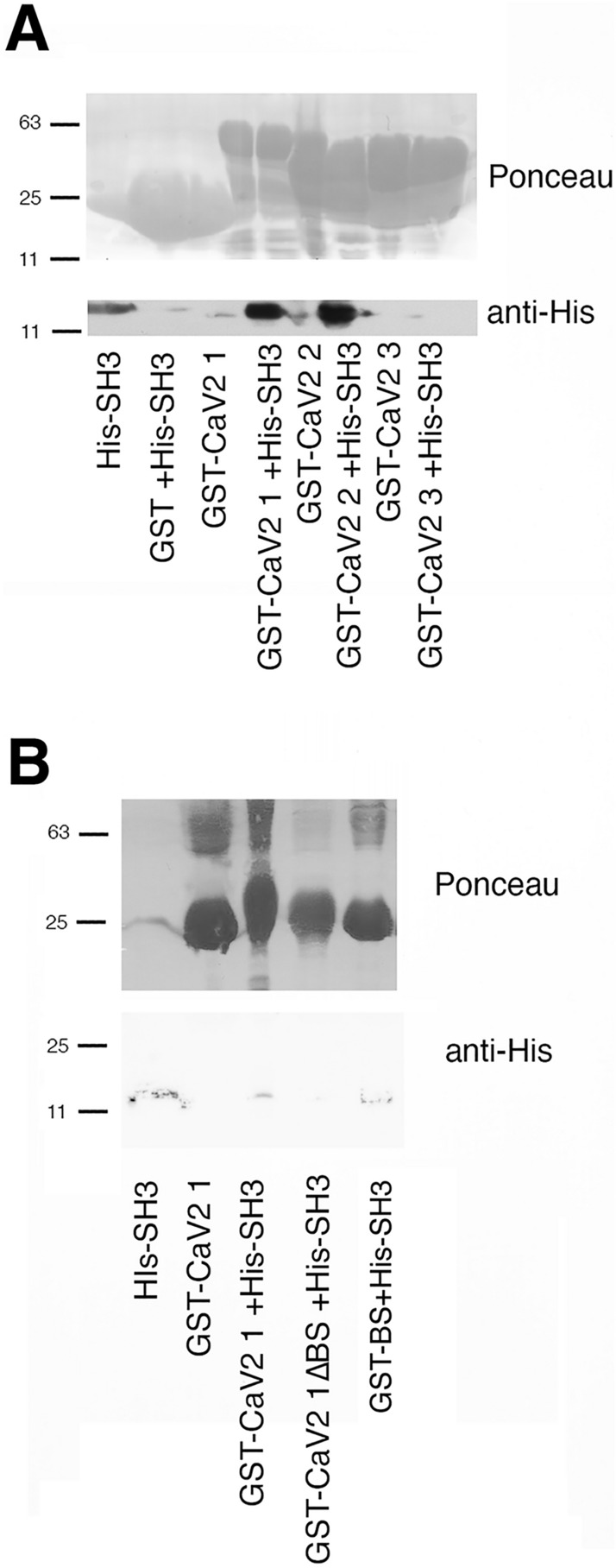
Ap RIM-binding protein binds the ApCa_V_2 alpha. (**A**) Pull down assay showing that the His-SH3-3 domain from Ap-RBP is selectively pulled down by ApCa_V_2a1 C-terminal fragments 1 and 2 (CaV2 1 and 2). Ponceau above shows the level of expression of the GST-ApCa_V_2a1 C-terminal fragments. Immunostaining with the anti-His protein shows the pull-down. 20% of the His-SH3-3 domain is loaded alone in lane 1. Supplemental Fig. 1 shows the entire blot. (**B**) Pull down assay identifying the RBP site in ApCa_V_2a1. His-SH3-3 was brought down by Ca_V_2 fragment 1, but not when the putative RxLPxxP site was removed. This site itself was sufficient to bring down the SH3-3 domain. Ponceau is shown above to show loading of the GST-ApCa_V_2a1 proteins and the SH3-3 was identified by blotting with the anti-His antibody. 20% of the His-SH3-3 domain is loaded alone in lane 1. Supplemental Fig. 2 shows the entire Ponceau stain and the entire blot.

There are 4 putative sites for the RBP SH3 domains that bind type I SH3 ligands (RxxPxxP) in the C-terminal of Ca_V_2a1, only one of which, however is predicted to bind to RBP using prediction algorithms^46^ and that contains the atypical leucine (RxLPxxP) important for RBP binding in vertebrates^47^(Supplemental Tables 4, 5). RBP has three SH3 domains, but SH3 domain 3 was the domain linked to function in *C. elegans*^44^, the domain shown to bind CaV2 in Drosophila^48^, and was the best expressing of the three domains from *Aplysia*. Thus, our studies focused on protein–protein interactions with the SH3-3 domain from RBP. We found that this domain, specifically bound to the first and second fragments of the ApCa_V_2a1 C-terminus, but not the third fragment which contains the most highly conserved RxxPxxP site, but one not predicted to bind to RBP (Supplemental Tables 4, 5) (Fig. 7A). Both the first and second fragment contains the predicted RBP site and indeed binding to the first fragments is largely reduced (26 + /- 16% of binding remaining, n = 4, SD, p < 0.01 one sample T-test) when this site is removed (Fig. 7B). Finally, this site in isolation was sufficient for RBP binding (Fig. 7B). Thus, the ApCa_V_2a1 C-terminal binds to both ApRIM and ApRBP. As well, ApRIM retains the RBP binding site between its two C2 domains (RxLPxxP) that is present in all bilaterian RIMs^35,47^, suggesting the conservation of the complex between RIM, RBP and Ca_V_2a1 in *Aplysia*. There are however, other highly conserved regions in the C-terminal (Supplemental Table 5) whose function is not known that may also contribute to the role of the C-terminal in localizing Ca_V_2 to synapses^39,49,50^.

## Discussion

The sensory neuron has relative differences in the expression of some, but not all alternative splices, compared to the total nervous system. This suggests differences in the levels of RNA binding factors that control splicing in sensory neurons. This is a common feature of neuron differentiation^51^. Indeed, differential splicing of Ca_V_2.2 in sensory neurons of vertebrates controls regulation by opioid peptides^52^. Alternate exon 27 is largely excluded from Ca_V_2 channels expressed in *Aplysia* sensory neurons. This exon appears orthologous to the vertebrate Ca_V_2.1 and Ca_V_2.2 alternate exon e24a, as all add a short 4 amino acid sequence to the domain III S3-S4 linker (SSTR in a1A, SFMG in a1B, and AFDS in a1 in *Aplysia*)^53^. In rodent DRG and SCG neurons, e24a inclusion is repressed in Ca_V_2.2 and the similar e31a (also a short insert in S3-S4 linker, but in domain IV) expression is enhanced under the transcription regulator Nova-2^54^. Whether a similar regulation leads to the prevalence of the exclusion of this exon from channels expressed in *Aplysia* sensory neurons is unclear. An ortholog to the vertebrate e31a does not appear in the *Aplysia* transcriptome, though *Drosophila* has an alternative exon in this place^55^. While conserved, the functional roles for this small insert are not known, the insert in domain IV has an effect on activation kinetics, while the insert in domain III does not^53^.

We examined two of the major differential splicing events in the sensory neurons, expressing the form not normally expressed in sensory neurons, but did not find effects on synaptic depression or reversal of depression. Moreover, our wildtype “WT” channel contains the shorter start site that is under-expressed in sensory neurons, but depression is not affected by expression of the WT channel^33^. It is not clear if the specific isoform of ApCa_V_2a1 expressed in sensory neurons is important for the function of sensory neurons or is just a non-selected feature of other splicing events required for sensory neuron function. In general, most of the splice sites examined are only seen in mollusks (Supplemental Table I) and are thus, likely not to regulate highly conserved synaptic regulatory events.

We found binding sites in ApCa_V_2a1 for both RIM and RBP. It has been proposed that Ca_V_2 localize to the presynaptic active zone through these two interactions^42,47,56^. While the DWC site is very well conserved, there have been no actual demonstrations of RIM binding to this site other than in vertebrates. In fact, Drosophila cacophony (ortholog of Ca_V_2a1) localize to synapses normally with the C-terminal putative PDZ ligand obstructed by GFP or other fluorescent proteins^57,58^. This may be result of the redundancy of these two interactions as reported in rodents^56^ but may also involve interactions with other synaptic proteins. The Ca_V_2a1 C-terminal PDZ ligand interacts with Mint1^59^ and this interaction was proposed to be important for Ca_V_2 function in another gastropod mollusc, *Lymnaea*^60^. Thus, our demonstration of RIM binding to this site is significant in demonstrating conservation of the direct Ca_V_2-RIM interaction, however, further examination of which interactions are physiologically relevant for the C-terminal ligand will be needed. The proximity of other conserved sites in the distal Ca_V_2a1 C-terminus further complicate the role of this highly conserved region of the protein^61^. We also observed a splice form of RIM that may act as a dominant negative, lacking binding to Ca_V_2a1 but retaining all other RIM interactions. This splice insert can also be seen in the related gastropod, *Biomphalaria,* but we have not observed this splice insert in any other molluscan transcriptome available on NCBI.

The RBP interaction with Ca_V_2a1 has also been characterized in *Drosophila*^48^, where it seems more crucial for transmitter release than in vertebrates^56^ or in *C. elegans*^62^. The site has been determined in *Drosophila* and, similar to *Aplysia*, there is a single site and it matches the consensus sequence RxLPxxP^48^. Here we provide further evidence as identification of this site in *Aplysia* and the confirmation of the conservation of RBP binding to Ca_V_2. RBP is an evolutionary ancient molecule, however, and was present before its partner RIM and before neurons evolved^34^ and so when it was recruited into its role in regulating Ca_V_2 is not clear. The Ca_V_2a1 RBP binding site, RxLPxxP in the C-terminus of the channel, appears in Ca_V_2 sequences from Cnidaria and Bilaterians but is not present in the available Placozoan sequence. The C-terminal PDZ ligand and the EF hand tyrosine involved in heterosynaptic inhibition also appear in Cnidaria and Bilateria, but not Placozoa suggesting these sites evolved soon after Ca_V_1 and Ca_V_2 diverged from a common high voltage-activated calcium channel. The conservation of these three regulatory sequences in the C-terminal of Ca_V_2 in Cnidarians is consistent with these interactions playing a role in the evolution of fast synaptic transmission in these organisms. The C-terminal of Ca_V_2a subunit is important in localization of the channel to the synapse, although no one binding site is necessary and sufficient for this role^39^. Expression of ApCa_V_2a1 is limited by specific cellular mechanism; overexpression does not increase the amount of channel expressed on the plasma membrane but leads to substitution of the channel on the membrane with the expressed form^33^. Moreover, expression is linked to a decrease in excitability presumably due to co-regulation of a potassium channel^33^. Neither of these features appears to require most of the C-terminal since overexpression of the short isoform of the channel starting immediately preceding the IQ domain acts similarly to the full-length channel in both respects (Fig. 4). However, the short isoform of the channel does not replace the endogenous channel at the synapse, as determined by the difference in the resistance to heterosynaptic inhibition when the Src site is mutated on a channel when the C-terminal is removed (Fig. 4). Importantly, no difference in resistance to heterosynaptic inhibition of the calcium influx at isolated sensory neurons was observed with expression of either the short or the long isoform, suggesting the short isoform replaces endogenous channels on the plasma membrane to a similar extent as expression of the full-length isoforms. The ability of the ApCa_V_2a1 Y-F short isoform to reduce heterosynaptic inhibition of the whole calcium current, but not the synaptic calcium current to a similar extant, indicates that the short isoform is unable to substitute or replace the endogenous, wildtype channels which presumably remain at the synapses and are subject to heterosynaptic inhibition. There was however, a trend for expression of the short channel to provide some relief of heterosynaptic inhibition, which may reflect contribution of the short isoform to the calcium microdomain at the synapse. Unlike some synapses, transmitter release at *Aplysia* sensory neurons is not triggered solely with calcium nanodomains, as transmitter release is sensitive to the slow calcium chelator EGTA^15^. Thus, even if the Ca_V_2a1 short isoform is excluded from synapses, there may still be some relief of heterosynaptic inhibition of the calcium current on transmitter release due to replacement of non-synaptic channels that partially contribute to the calcium triggering transmitter release. The implication that wildtype channels remain at the synapses with expression of the short ApCa_V_2a1 isoform prevents assessment of a potential role for the loss of most of the C-terminal in depression and reversal of depression as the wildtype channels presumably remain to trigger release. Further studies will be required to definitively determine if the interactions of ApCa_V_2 with active zone elements plays a role in synaptic depression or reversal of depression.

Ca_V_2 alpha1 subunits with complete C-terminal truncations have been reported to express but lack surface expression and contribution to the calcium current^39^. However, inclusion of the proximal C-terminus leads to surface expression of functional channels^49^. Similarly, splicing in Ca_V_2.2 channels that removes the C-terminal RBP and RIM bindings sites reduces synaptic localization but not surface expression^63^. Our ApCa_V_2a1 short isoform retains a significant portion of the early C-terminus which includes the EF-hand and the pre-IQ domain, and when expressed contributes to the calcium current suggesting surface expression of a functional calcium channel. Heterosynaptic inhibition is mediated by a Src site in the EF hand, and thus inclusion of this domain was necessary for us to measure whether the channel was expressed on the membrane. Inclusion of these key domains may allow for the surface expression of the ‘short’ channels expressed here as compared to others.

## Materials and methods

### Cloning strategies

DNA sequences encoding for the PDZ domain of Aplysia RIM, Aplysia RIM splice, ApCa_V_2a1 C-terminal domain or C-terminal domain fragments 1–3, SH3 domain 3 of Aplysia RBP protein were purchased as G blocks from Integrated DNA technology (IDT) or generated by PCR. All resultant amino acid sequences and NCBI IDs are listed in Supplemental Table 5 and all PCR primers are listed in Supplemental Table 2. The G blocks or PCR products were initially cloned in to pJET (Thermofisher K1231) using instructions with the kit and the sequence confirmed. The insert was then excised with BamHI and EcoRI, and for the Ca_V_2 C-terminal domain, Ca_V_2 fragments or SH3 binding site, inserted into a vector encoding GST fusion proteins (PGEX-2 T (GENBANK A01438); or for PDZ and SH3 domains, a vector encoding His-tagged fusion protein (PTrcHisB; Thermofisher V36020). The constructs were transformed into DH5α E. coli cells.

### Purification of fusion proteins

DH5α cells were grown in Luria Broth (LB) media with ampicillin (50 μg/mL). Protein expression was induced at an OD600 of 0.28–0.32 with Isopropyl β‐D‐1‐thiogalactopyranoside (IPTG) (0.1 mM) for 2 h at 37 °C, and cells were centrifuged for 10 min at 3100 g at 4 °C. They were then resuspended in lysis buffer (PBS, 1.0 mM PMSF, 1% Triton-X-100, 02.5 mM B- mercaptoethanol, 1 Roche Complete Protease Inhibitor Cocktail tablet), sonicated 6 × 10 s, and centrifuged at 10,000 *rpm* for 30 min. The supernatant from the GST protein lysate wasincubated with Glutathione Sepharose 4B beads (GE Healthcare) overnight at 4 °C, while the supernatant of Histag proteins was incubated with His-Select Nickel Affinity GelProbond (Sigma-Aldrich) for 1 h at 4 °C on a rotator. Unbound protein was washed 4 × 10 min with PBS. The His-tag proteins were eluted off the beads with elution buffer (PBS, 250 mM Imidazole), while the GST proteins were kept on the beads. GST fusion proteins were stored at 4 °C for no longer than 5 days before use, and His-tag proteins were used fresh.

### GST pulldown assay

GST protein on beads were rotated with His-tag protein eluate overnight at 4 °C. Before the pulldown, the His-tag protein eluate was quantified using the DC™ Protein Assay (Bio-Rad)to ensure the presence of approximately equal amounts of His-tag protein across all pulldowns within each experiment. Unbound His-tag protein was washed 4 × 10 min with PBS. Theproteins were eluted off the beads by boiling in Laemmli sample buffer at 95 °C for 5 min and separated by sodium dodecyl sulfate–polyacrylamide gel electrophoresis (SDS-PAGE). The resulting gels were stained in Coomassie (0.25% Coomassie Brilliant Blue R-250, 30% Methanol, 10% Glacial Acetic Acid) or transferred to a nitrocellulose membrane (0.45 μm, Bio-Rad) (24 V for 90 min) for Ponceau- and immuno-staining.

### RT-PCR

Isolated sensory neurons or nervous system ganglia were frozen on dry ice and total RNA was isolated^64^. RNA was reverse transcribed from each of these groups into cDNA using the Superscript II Reverse Transcriptase (Invitrogen) and following the kit protocol. This was the template for PCR reactions. For semi-quantitative PCR reactions, Temperature and cycle number were adjusted in each case to allow detection but avoid saturation.

### Cell culture and plasmid microinjection

*Aplysia Californica* were obtained from the National Resource for *Aplysia* at the University of Miami and housed in holding tanks at 16C. *Aplysia* central ring ganglia were digested with dispase and ventrocaudal cluster pleural sensory neurons were cultured in isolation or paired with abdominal ganglia LFS motorneurons overnight at 19C on glass bottomed poly-L lysine coated dishes in culture media composed of L-15 modified with *Aplysia* salts, *Aplysia* hemolymph (from 10 to 50%), and supplemented with L-glutamine. Following 24 h in culture, sensory neurons were pressure injected with glass pipettes filled with pNEX3 plasmids using a WPI picopump and left in culture for a further 48 h to allow for Ca_V_2 surface expression^33^. Vectors were injected at the following concentrations in ug/uL, pNEX3-GCaMP6f 0.2, pNEX3-5HT1a 0.2, all pNEX3-RFP-ApCa_V_2a1 isoforms 0.6.

### Live cell fluorescence imaging

Wide field epifluorescent imaging was conducted on a Zeiss Axioobserver D1 with a EC Plan Neofluar 40 × 1.3NA lens and a QuantEM:512SC EM-CCD camera (Photometrics). A Zeiss SVB-1 microscope signal distribution box and Axiovision software was used to acquire GCaMP6f fluorescence through Zeiss GFP 38 filter cube at a ~ 50 ms frame rate. Single action potential transients were evoked with sharp electrodes and measured at regions of interest selected as sites of action potential calcium entry before application of 8-OH-DPAT.

Background corrected fluorescence values from the regions of calcium entry were converted into a DF/Fo value using the measurements from the ten frames preceding the action potential. The peak of three consecutive action potential induced transients were measured and averaged before and after application of 8-OH-DPAT.

### Electrophyisology

Cells were impaled with bridge-balanced sharp glass electrodes backfilled with 2 M potassium acetate (~ 15MΩ) connected to an Axoclamp 900 amplifier, Digidata 1400 digitizer, running pClamp acquisition software (Molecular Devices). Prior to recording the culture media was replaced with normal saline [in mM: 460 NaCl, 55 MgCl_2_, 10 CaCl_2_, 10KCl, 10 D-Glucose, and 10 HEPES pH 7.6]. Cells were held at − 80 mV with current injection and single action potentials generated with 20–50 ms depolarizing pulses. The intensity of the depolarizing pulse varied from cell to cell and the intensity initially set to subthreshold and increased to produce a single, isolated action potential. At synaptic connections between sensory neurons and motorneurons, 40 action potentials were generated in the presynaptic sensory neuron at 0.05–0.1 Hz to depress the synapse (homosynaptic depression HSD), then 10 μM 5HT applied and another 10–15 action potentials evoked to measure the reversal of synaptic depression (R-HSD)^65^ Postsynaptic potential (PSP) amplitude or initial rise-rate was measured for all stimuli using Clampfit and analyzed with Excel and Prism.

For experiments involving Ca_V_2 expression, recordings were only used if the sensory neuron injected had clear RFP expression and displayed the characteristic change in membrane excitability reported previously, which requires at least 48 h expression of RFP tagged Ca_V_2 channels^33^.

### Statistics

Statistical comparisons were made in Prism, error bars and means are ± standard error of the mean. Specific tests are described when used.

### Supplementary Information

Supplementary Information.

## Data Availability

All data and constructs used in this paper are freely available. Please contact the corresponding author Wayne Sossin at wayne.sossin@mcgill.ca.
