# Supplementary material for: The role of specific isoforms of CaV2 and the common C-terminal of CaV2 in calcium channel function in sensory neurons of Aplysia
Source: Sci Rep. 2023 Nov 18;13:20216. doi: 10.1038/s41598-023-47573-z (PMC10657410; doi:10.1038/s41598-023-47573-z)
Supplement: Supplementary file 1 — Supplementary Information. [file 41598_2023_47573_MOESM1_ESM.pdf]

Supplemental Figure S1

Fig. 1b left

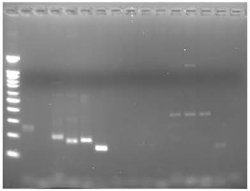

Fig. 1b right

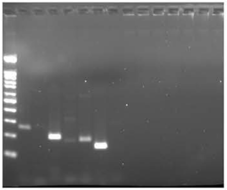

Figure 2A AND Figure 2B

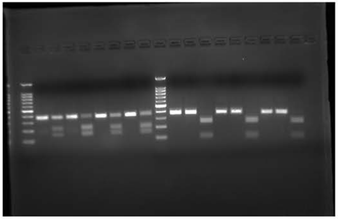

Figure 2C

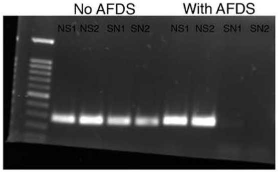

Figure 2D

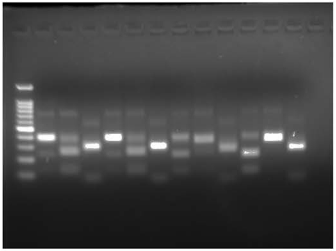

Figure 2E

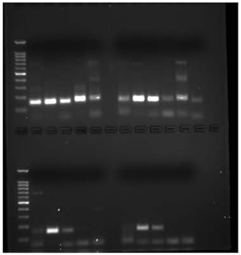

Fig. 3A

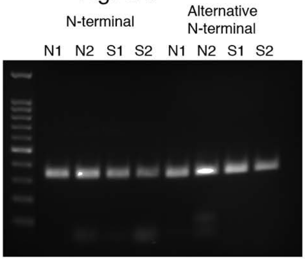

Fig. 3B

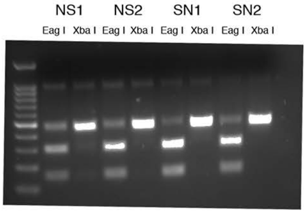

Figure 6C

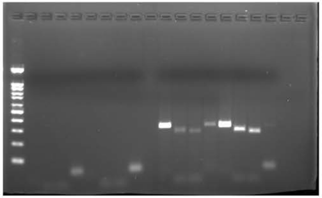

Supplemental Figure 2

Figure 7B

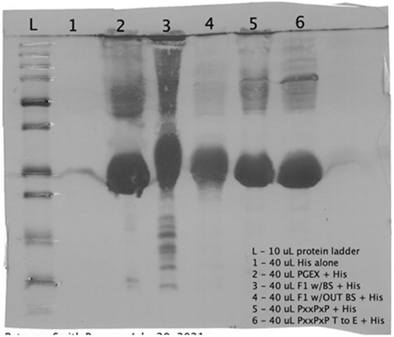

Figure 7A

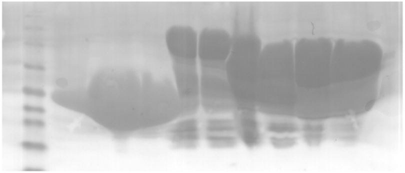

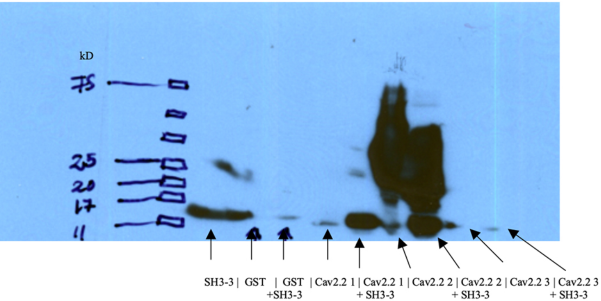

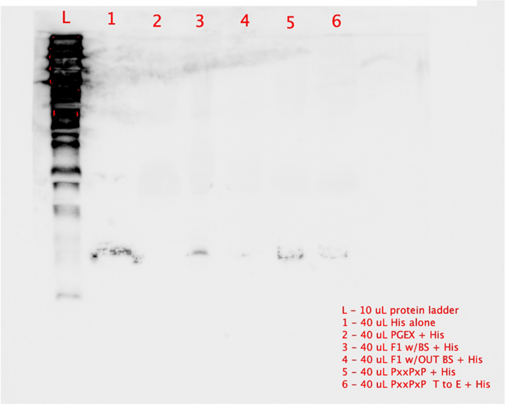

Supplemental Figure Legends

.

Supplemental Figure 1. Uncropped Agarose Gels for Figures, 12,3 and 6. Uncropped gels are shown with the corresponding Figure shown above. For Fig. 1b left, right, the gel was cropped to remove unrelated experiments on the left side of the gels. For the gel for Figure 2A AND Figure 2B, the left side is Fig. 2A and the right side is Fig. 2B. For Figure 2E the left side top (NS) and bottom (SN) are used in the figure and the right side is the same experiment with an independent NS and SN library. The sixth lane in the right half of the gel represented a PCR primer pair that is not discussed in the paper. For Figure 6C, the left side represented the same experiment using a library that lacked the concentration required for this experiment. For all other figures, all lanes are shown in the paper, but an expanded view of the agarose gel is shown.

Supplemental Figure 2. For Figure 7A, the uncropped Ponceau blot is shown on top and the entire film is shown on the bottom. In the figure the written arrows were cropped from the bottom. The large smears ween for GST Cav2.2 are due to a stretch of histidines in the sequence that cross-reacts with the anti-His antibody used for thsis experiment. For Figure 7B, the uncropped gels are shown. Lane 6 is not shown in the figure in the paper as we did not present data using this construct in the paper.

Supplemental Table S1 Exons of ApCaV2a1

Exon	Genomic Start	Genomic End	Intron length between CDS exons (bp)	Intron length between 5'UTR and CDS	CaV2 region	Conservation	ing transcript	Alt Splice	Comments
UTR-A1	1253149	1252796		68655			Trinity_DN4245_c1_g3_i2.DNA		
1	1184141	1183424			N-terminal	BiV	Trinity_DN4245_c1_g3_i2.DNA		
UTR-B1	1159828	1160134		110498			Trinity_DN4245_c1_g3_i3.DNA		
2	1049636	1049377	133788		N-terminal	M	Trinity_DN4245_c1_g3_i3.DNA		
UTR-C1	991084	990770		50151			Trinity_DN4245_c1_g3_i4.DNA		
UTR-C2	950986	950916		10297			Asmbl_140481		Unigene
3	940619	940418	108758		N-terminal	M	Asmbl_140481		
UTR-D	950986	950916		10297			Asmbl_140532		
4	902712	902607	37706			BiV	Asmbl_140481		
5	901543	901401	1064		ion channel repeat I	CaV2	Asmbl_140481		
6	895379	895273	6022		ion channel repeat I	CaV2	Asmbl_140481		
7	890054	889887	5219		ion channel repeat I	U	Asmbl_140481		
8	888544	888329	1343		ion channel repeat I	U	Asmbl_140481		
9	886158	886055	2171		ion channel repeat I	U	Asmbl_140481		
10	885211	885095	844		I-II linker	U	Asmbl_140481		
11	884393	884334	702		I-II linker	U	Asmbl_140481		
12	882164	882057	2170		I-II linker	CaV2	Asmbl_140481		
13	881193	880983	864		I-II linker/ ion channel repeat II	U	Asmbl_140481		
14	878459	878345	2524		ion channel repeat II	U	Asmbl_140481		
15	877088	876978	1257		ion channel repeat II	CaV2	Asmbl_140481		

16	876004	875869	974	ion channel repeat II	U	Asmbl_140481	
17	875317	875246	552	ion channel repeat II	U	Asmbl_140481	
18	874673	874550	573	ion channel repeat II	U	Asmbl_140481	
19	873018	872918	1532	II-III linker	CaV2	Asmbl_140481	
20	869952	869943	2966	II-III linker	M	Trinity_DN256_c0_g1_i4	DDL
21	862016	861888	7927	II-III linker	G	Asmbl_140481	
22	860280	860120	1608	II-III linker	G	Asmbl_140481	
23a	857734	857704	2386	II-III linker		Asmbl_151519	CWRARIGFRI
23b	854343	854309	3361	II-III linker		Asmbl_140532	NHKPGAVLG
24	850431	850296	3878	II-III linker/ ion channel repat III	CaV2	Asmbl_140481	
25	849475	849284	821	ion channel repeat III	U	Asmbl_140481	
26	848618	848513	666	ion channel repeat III	U	Asmbl_140481	
27	844549	844571	3964	ion channel repeat III	Bil	Trinity_DN268898_c5_g5_i7	AFDS
28	838612	838512	5959	ion channel repeat III	U	Asmbl_140481	
29	837110	836930	1402	ion channel repeat III	BIl	Asmbl_140481	
30	836014	835871	916	ion channel repeat III	Bil	Asmbl_140481	
31	834359	834155	1512	III-IV linker	Bil	Asmbl_140481	
32	833240	833075	915	ion channel repeat IV	Bil	Asmbl_140481	
33a	826978	827082	6097	ion channel repeat IV	Biv	Asmbl_140482	alt TM region
33b	819557	819418	7525	ion channel repeat IV	Biv	Asmbl_140481	
34	809892	809808	9526	ion channel repeat IV	U	Asmbl_140481	

35	809105	808988	703	ion channel repeat IV	U	Asmbl_140481		
36	807795	807727	1193	ion channel repeat IV	U	Asmbl_140481		
37	807081	806991	646	ion channel repeat IV	U	Asmbl_140481		
38	806267	806102	724	ion channel repeat IV	U	Asmbl_140481		
39	804033	803902	2069	ion channel repeat IV/EF hand (GPHH domain)	U	Asmbl_140481		
40	803225	803125	677	EF hand (GPHH domain)	U	Asmbl_140481	Src phosphorylation site	
41	801589	801483	1536	pre-IQ	CaV2	Asmbl_140481		
42	799027	798919	2456	preIQ	CaV2	Asmbl_140481		
43	798386	798276	533	IQ	CaV2	Asmbl_140481		
44	791941	791921	6335	C-TERMINAL	CaV2	Asmbl_140481		
45	782811	782796	9110	C-TERMINAL	Ap	Asmbl_140481	DPVRLPK	asmbl_151398 (lacking exons 45-48)
46	775814	775670	6982	C-TERMINAL	M	Trinity_DN256_c0_g1_i7	NHNEK	
47	768433	768319	7237	C-TERMINAL	M	Asmbl_140481	large alt splice	includes repeat of 20 Glycines
48	763680	763627	4639	C-TERMINAL	M	Asmbl_140481	alt splice	
49	754063	753754	9564	C-TERMINAL	G	Asmbl_140481		
50	752102	743056		C-TERMINAL AND 3'UTR	G	Asmbl_140481	7.8 KB OF 3'UTR	

The number and position of each exon is shown. Numbers are based on the Hi-C Scaffold genome on http://aplysiatools.org:8080/. All sequences are on scaffold 12.
Conservation Ap, Aplysia, Bil, Bilaterian,BiV, Bivalve G, gastropod; M, Mollusc, CaV2 (present in Cnidarian CaV2), U, Universal (present in CaV1 as well). Exons were examined in selected species with genome resources.

Supplemental Table S2: PCR primers used

Site being tested	Forward primer	Reverse Primer	Size (bp)	Unique restriction enzyme
CaV2.2 alpha				
Start site				
A vs B				
A2	CGCTACACGGCCGGATATATC	TTGCGAGCTTTGTAGCGGAG	260	
B1	TCTGTGGGAGCAGAAGCTGA	TTGCGAGCTTTGTAGCGGAG	273	
AB vs C				
B2	TCGGATTTACGCACGTGCGC	ACAGGGACCGGGTGCTGTTG	204	
C1A	TTTGGGCCTTCTGCGCATGT	ACAGGGACCGGGTGCTGTTG	185	
C1B	CCGGCATGGAGCACGGATAG	ACAGGGACCGGGTGCTGTTG	190	
ABC vs D				
C2	GGTATGGCTGCCAACAGCAC	CCTCCAGGGCCAGGACTATAC	150	
D1	GGTTCTACCCGGTGCCATG	CCTCCAGGGCCAGGACTATAC	150	
Exon 20 (DDL)	GAGAAGAGTGGCATGCTG	CCACGGAGATCTTCTTGTC	330	PsHA1
Exon 23 (a/b)	GCTCGTTAACATCTGTCCTCCC TC	TTGGGTTTGTCGGGTAAAAGA TG	390	a:MvAI<br>b: AvrII<br>Both: NcoI
Exon 27 (AFDS)				
No exon	TCAACCTCCGCTACTTCGATC	TACCCGCGCTCTCATTAAAG	380	
Exon	TCAACCTCCGCTACTTCGATC	TACCCGCGCTCTCACTATC	380	
Exon 33 (a/b)	CTGTGTCGGTTCATGCCCAAG	CGGAAAAACCCGAAGCTGACT C	340	a:AgeI<br>b: FspI<br>Both:XmnI
Exon 45-48				
45	TCGGAAACTGATCCGGATGA	AGACGGACGGGGTCCATT	190	
46 (no 45)	TCGGAAACTGATCCGGATGA	GGTCTCTTTTCGTTGTGGTTCA TT	190	
45+46	TCGGAAACTGATCCGGATGA	TCGTTGTGGTTCTTGGGCAG	210	
No 45 NO 46	TCGGAAACTGATCCGGATGA	GGAACAGAGACGGTCTCATT	175	
No 45-48)	TCGGAAACTGATCCGGATGA	GAAAAGTCGTTGCCGGGCATT	170	

CaV beta start				
A	CTCGCCCCAACTCAGTTTCAG	GCATAGCCGTGTACCGGT	350	
B	ATGCGCATCAAAACAGAAGGT	GCATAGCCGTGTACCGGT	350	
CaV alpha2-delta	CGGTCCGTGTGTTTACCTAC	CCACATTGGGCGGTTCTTTC		A: EAGII B) XBA
	CCAGAGAAACAGACGATGGACGA	GATGCACGACGGAGAATGAAGA	300	MluI
RIM family				
RIM	GCCTCTCAAAGAGAATGGCA	CCACCAATCACTTTCAGACC.		
RIM Δ splice	GCCTCTCAAAGAGAATGGCA	TCAGACCTAACGCTTCAGAC.		
Piccollo	GATTGTTGGCGGTAAGGAGA	ACGACGGAAGTACCAGGTTG		
Fife	TCGTCTTCACTTTGCACCAC	GGCTTCATGACTTCCTCTGG		
Cloning primers				
Remove site from CaV2a1 fragment 1	gggggatccggcaacgacttttcggga	cccccgaattcctagggtgacggtgaccttga		
Just Sh3 site	gatccatgacgaaaagagcctcacgtcggctacctgtggcgccctctccctagG	aattcctagggagagggcgccacaggtagccgacgtgaggctcttttcgtcatG		

Nucleotides for all PCRs are shown.

Supplemental Table S3: Additional information of PCR to determine exons.

AFDS

```
Ala Phe Asn Ala Phe Asp Ser Glu Ser Ala Gly Lys Asn Leu Asn
GCC TTT AAT GCC TTT GAT AGT GAG AGC GCG GGT AAG AAC CTG AAC
               ←----------------------------

Ala Phe Asn Glu Ser Ala Gly Lys Asn Leu Asn
GCC TTT AAT GAG AGC GCG GGT AAG AAC CTG AAC
  ←-----------------------
```

```
Alt exon in TM segment 4

Leu Asn Thr Iso Val Leu Met Met Lys Phe Asp Pro Lys Glu Ser Arg Gln Ser
CTC AAC ACC ATC GTG CTC ATG ATG AAG TTT GAC CCC AAG GAA TCT CGC CAG TCC

Arg Arg Arg Glu Lys Gly Glu Asp Ala Ala Arg Iso Leu His Leu Iso Asn Thr
CGG CGC CGT GAG AAG GGG GAG GAC GCG GCC CGC ATC CTC CAT CTC ATT AAC ACG

Val Phe Thr Ser Leu Tyr Gly Leu Gly Phe Leu Leu Lys Leu Cys Ala Tyr Gly
GTC TTC ACC TCC CTC TAT GGC CTG GGG TTT CTG CTC AAG CTA TGC GCA TAC GGA
                                                         FSPI
Lys Asn Tyr Phe His Asp Pro Trp Asn Val
AAG AAT TAC TTC CAC GAC CCC TGG AAT GTG
       XMN I

Leu Asn Thr Iso Val Leu Met Met Lys Tyr Glu Thr Ser Asp Thr Tyr Lys Glu
AAC ACC ATC GTG CTC ATG ATG AAG AAA TAT GGC ACG TCG GAC ACA TAT AAG GAA

Val Leu Lys Tyr Leu Asn Ala Gly Phe Thr Ala Leu Phe Thr Iso Glu Cys Thr
GTC CTG AAA TAC CTA AAC GCA GGC TTT ACG GCT TTG TTC ACC ATA GAG TGT ACC

Iso Lys Iso Leu Gly Thr Gly Ala Arg Asn Tyr Phe His Asp Pro Trp Asn Val
ATC AAG ATA TTA GGC ACC GGT GCC AGG AAT TAC TTC CAC GAC CCC TGG AAT GTG
                     AGE I               XMN I
```

```
Trp Lys Ala Tyr Lys Ala Ser Gln Asn Ala Ser Asn Asn Phe Lys Met Arg Pro
TGG AAG GCT TAC AAG GCC AGC CAG AAC GCT AGC AAC AAC TTC AAA ATG AGA CCG
                                                          ←-----------

Ser Leu Phe Arg
TCT CTG TTC CGG
-------------


Trp Lys Ala Tyr Lys Ala Ser Gln Asn Ala Ser Asn Asn Phe Lys Met Asp Pro
TGG AAG GCT TAC AAG GCC AGC CAG AAC GCT AGC AAC AAC TTC AAA ATG GAC CCC
                                                          <------------

Val Arg Leu Pro Lys Arg Pro Ser Leu Phe Arg
GTC CGT CTG CCC AAG AGA CCG TCT CTG TTC CGG
----------


Trp Lys Ala Tyr Lys Ala Ser Gln Asn Ala Ser Asn Asn Phe Lys Met Asn His
TGG AAG GCT TAC AAG GCC AGC CAG AAC GCT AGC AAC AAC TTC AAA ATG AAC CAC
                                                          ←-----------

Asn Gly Lys Arg Pro Ser Leu Phe Arg
AAC GAA AAG AGA CCG TCT CTG TTC CGG
------------------


Trp Lys Ala Tyr Lys Ala Ser Gln Asn Ala Ser Asn Asn Phe Lys Met Asp Pro
TGG AAG GCT TAC AAG GCC AGC CAG AAC GCT AGC AAC AAC TTC AAA ATG GAC CCC

Val Arg Leu Pro Lys Asn His Asn Glu Lys Arg Pro Ser Leu Phe Arg
GTC CGT CTG CCC AAG AAC CAC AAC GAA AAG AGA CCG TCT CTG TTC CGG
        ←------------------------


Trp Lys Ala Tyr Lys Ala Ser Gln Asn Ala Ser Asn Asn Phe Lys Met Pro Gly
TGG AAG GCT TAC AAG GCC AGC CAG AAC GCT AGC AAC AAC TTC AAA ATG CCC GGC
                                                          ←-----------

Asn Asp Phe Ser Gly Gly
AAC GAC TTT TCG GGA GGA
--------------


Splice           WKAYKASQNASNNFKM------------RP (large splice) PGNDFSGG
DPV + Splice     WKAYKASQNASNNFKMDPVRLPK-----RP (large splice) PGNDFSGG
NHN + Splice     WKAYKASQNASNNFKM-------NHNEKRP (large splice) PGNDFSGG
DPV NHN +Splice  WKAYKASQNASNNFKMDPVRLPKNHNEKRP (large splice) PGNDFSGG
No Splice        WKAYKASQNASNNFKM------------------------------PGNDFSGG
```

Supplemental Table S4. Amino acid sequences of constructs used in this study.

ApRIM constructs

RIM-PDZ XP_035827827.1
HPVTWQSSADGTKWIGHMILKKTVLEGSGEKRDSSAILGLKVIGGKVSDSGKLGAFITKVKKGS
IADTVGHLRPGDEVVEWNGRSLQGATFDEVYDIILESKQEPQVELIVHR*

RIMSpl-PDZ XP_035827829.1
HPVTWQSSADGTKWIGHMILKKTVLEGSGEKRDSSAILGRSEALGLKVIGGKVSDSGKLGAFIT
KVKKGSIADTVGHLRPGDEVVEWNGRSLQGATFDEVYDIILESKQEPQVELIVHR*

ApCaV2a1 constructs

CaV2a1 C-terminal short
WPVQGKKMMDLLMPPSDES*

GST-CaV2a1 C-terminal AVD53847.1
VILPNGFKPKGRKPEKYEMRTDSNTALKEDSDEDDDDWC*

GST-CaV2a1 C-terminal ΔPDZ recognition site
VILPNGFKPKGRKPEKYEMRTDSNTALKEDSDEDD*

CaV2a1 C-terminal *Fragment #1* AVD53847.1
GNDFSGGLRPEHASALNSRAELGGRGGARSPSLPPTPLSPRSPMGGAQSPFGSPRASPVP
SRRSPSPRRFDVGFAAAVANLCEQAHNIADQDRQRKYGMKTEDSISSSSPTFRGRSRQRP
RPPLQSQSPVLGSPLPSPAHPRARVAGDSGFYRSTSLETRSRSPSPNLTASPPPRSGSTSLI
QRSRSPSPSAAAGSPPMTKRASRRLPVAPSPTGHGSV*

CaV2a1 C-terminal *Fragment #2* AVD53847.1
SGSTSLIQRSRSPSPSAAAGSPPMTKRASRRLPVAPSPTGHGSVGAGGGGGHHHHPSSST
SSPAKPASLNLTEPRYRSGDGRGMSHVEKDTLPVRGVPSPPSTRGGNINFPRLNASPTRV
PKLN*

CaV2a1 C-terminal *Fragment #3* AVD53847.1
DGRGMSHVEKDTLPVRGVPSPPSTRGGNINFPRLNASPTRVPKLNIPVSSSSQPMTSSSSR
HAHPPSSASASSQHHPHPQAPPPGRLGRPEPYSPTERNNLNKMSDPSRSSTLPAAHRTSG
YTRD*

CaV2a1 C-terminal *Fragment #1ΔRBP site*GNDFSGGLRPEHASALNSRAELGGRGGARSPSLPPTPLSPRSPMGGAQSPFGSPRASPVP
SRRSPSPRRFDVGFAAAVANLCEQAHNIADQDRQRKYGMKTEDSISSSSPTFRGRSRQRP
RPPLQSQSPVLGSPLPSPAHPRARVAGDSGFYRSTSLETRSRSPSPNLTASPPPRSGSTSLI
QRSRSPSPSAAA*

CaV2a1 C-terminal #1 RIM-binding protein site alone
MTKRASRRLPVAPSP*

ApRIMBP construct

RIM BP SH3-3 XP_012940834.1
KMIAMYDYDPQELSPNVDAELELSFKTGDTVLIYGDMDEDGFFTGEVNGQHGLVPSNFLQPA*

The amino acids used in study with the reference sequence from which the amino acids were derived. The splice region in the RIM=PDZ splice sequence is underlined as is the putative RIM-binding protein (RBP) site in ApCaV2a1 Fragment #1 and #2.

Commented [t1]: This should be ApCaV2a1 or CaV2a1, also above where highlighted

Supplemental Table S5. Alignment of the carboxy-terminal of Molluscan CaV2 channels

```
       {Fragment 1 start

OB      --DFSWGLRPEHAAHIPAPRPGTGSSKGLLFTHTAVPLSPVSPRSPL----PPQSPLGSP 54
MY      --DFSRGLKPEHAGHVSMSRTNSDQSIRGGSKSPSLPGTPVSPRS---PLMPHRSPFGSP 55
MG      --DFSSGLKPEHASGQG-----PTAPSRPGSKSPSVPTTPVSQRSPIHPMMPYHSPFGSP 53
PC      --DFSRGLRPEHAGHQSAPRMTPDNHQRSGNRSPSLPTTPVSPRSPL---GSIHSPYGSP 55
AC      GNDFSGGLRPEHASALNS---RAELGGRGGARSPSLPPTPLSPRSPM---GGAQSPFGSP 54
LS      GTDFSGGLRPEHASSLTS---R---GDRAGARSPSLPPTPLSPRSPM---G-AQSPFGSP 50
BG      GADFSGGLRPEHASSFIG---R---GDRSGARSPSLPTTPMSPRSPM---G-AASPFGSP 50
          *** **:****.                    ::* :*:* **         ** ***


OB      MASPSMHRRSVSPRRGLDVGFASAVSNIVDQAHSIAEHERHRKHRGGYF-YEGKHDDSLS 113
MY      VGSPYMGRRSPSPRR-VDLGFASAVTNICEQAHSIAEQDRRSKHYRNRDYEGILLDETLS 114
MG      TNSPMLQRRSPSPRR-NEFGFASAVSNLVDQAHSIAEMDRRK-------HYGFKADESLS 105
PC      RASPVPSRRSPSPRR-FDVGFAAAVTNLVEQAHTIADQDRRK-------PYVAKHEDSLS 107
AC      RASPVPSRRSPSPRR-FDVGFAAAVANLCEQAHNIADQDRQR-------KYGMKTEDSIS 106
LS      RASPIPSRRSPSPRR-FDVGFASAVANLCEQAHTIADQDRQK-------RYGVK-EDSIT 101
BG      RASPVPTRRSQSPRK-FDVGFAAAVTNLCEQAHSIADQDRQK-------RFGMK-EDSIT 101
          **   *** ***:  :.***:**:*: :***.**: :*:              :::::


OB      VPTSPQMRGRSRGRHRPPLQQQGQILGSPLPSPTAR-----RKEPTFYRSTSLENRSRSP 168
MY      LPNSPQMR--GRSKTRPPLTMQSNVIGSPAPSPQPL----RKRDTTFYRSTSLETRSRSP 168
MG      LPNSPQQRK-SRSLRRPPLQPQGNVIGSPLPSPQPL----RKRDSAFYRSTSLETRSRSP 160
PC      ---SPQLRG--RSRKRPPLQTQSPVLGSPLPSPGPMHRGKGREERGFYRSTSLETRSRTP 162
AC      S-SSPTFRGRSRQRPRPPLQSQSPVLGSPLPSPAHP-RARVAGDSGFYRSTSLETRSRSP 164
LS      S-SSPTFRGRSRQRSRPPLQAQSPVLGSPLPSPAHP-RVRGGGDSGFYRSTSLETRSRSP 159
BG      S-SSPTFRGRSRQRSRPPLQSQSPVLGSPLPSPGLP-RLRNMGDSGFYRSTSLETRSRSP 159
           **  *  .*   ****  *. ::*** ***          :  ********.***:*

                       {Fragment 2 start          Predicted RBP binding.

OB      SPNLTPTPMLHQHEYYGSANLTDRSRSPSPGPTVT---QPRKATRKLPAVPS-------- 217
MY      SPT-TTPSQTPQQEYYGTANLTDRSRSPSPAVTSP-PK---KPGRKLPSIPP-------- 215
MG      SPSTTTPSQTPQAEYYGTANLTDRSRSPSPVSTPP-KKQKQKQGRKLPPVPL-------- 211
PC      SPNTGHS--PSQQEYYGSTNLIDRSRSPSPSPAQSPPKRT---PRKLPPVPFVPST---- 220
AC      SPNLTAS--PPP--RSGSTSLIQRSRSPSPSAAAGSPPMTKRASRRLPVAPSPTGHG--- 217
LS      SPNLTAS--PPP--RSGSTSLVQRSRSPSPSLVAASPPMTSSAHRRLPVAPSSTSSS--- 212
BG      SPNLTAS--PPL--RSGSTSLIQRSRSPSPSMIAASPPMINRQHRRLPMAPSSTSSS--- 212
        **.             *::.* :*******              * **  *

              }Fragment 1 end                  {Fragment 3 start

OB      --------------------------KPSTLNLAQTRPRD-NMPRVMPSP--------TV 242
MY      --------------------------KPSSLNLAQPKLKD-NMPRVMPSP--------TI 240
MG      ----------------------PPALKPSTLNLSTPKLKDKNLPRVMPSP--------TI 241
PC      PPGMGPMPSTPVQVSGRGSSSSGPVTKPATLNLTQPKLKE-TMPRVMPSP--------TV 265
AC      ----SVGAGGGGGHHHHPSSSTSSPAKPASLNLTEPRYRS-GDGRGMSHVEKDTLPVRGV 272
LS      ----GSG-----------VT-TMSPAKPVSLNLSEPRYRD-NI------AIKDTLPVRGA 249
BG      ----GSG-----------TTTTTSPAKPVTLNLSEPRYRN-NI------AIKDTLPVRGA 250
                                  ** :***:  : :.
```

```
                                    }Fragment 2 stop

OB        PQPS-KSPGSINFPRLNASPTHIPRIGPTVGQ---------------------------- 273
MY        PQPP-KSPGSINFPKLNKSPTHVPKQNVPLTS---------------------------- 271
MG        PQPP-RSPGNINFPRLNQSPSHAPRNFPSSGSY--------------------------- 273
PC        PQAP-KSPSSINFPRLSTSPTRLQKNSDPKAGVAGAHSHHNQNRSAAATAGSVHQAPPLS 324
AC        PSPPSTRGGNINFPRLNASPTRVPKLNIPVSSSSQPMTSSSSRHAHPPSSASASSQHHPH 332
LS        PSPPG--RGNINFPRLNASPTRVPKLNIPVSSTSGIAAA-------------SPLPPHHH 294
BG        ISPPG--RGNINFPRLNASPTHVPKLNIPVSATSSGTAV-------------APIPPHHV 295
           .      ..****:*. **::  :


                                                            }Fragment 3 stop
OB        --APPLGRLGRPEPYSPTERNSLSKLSPE-----RSRTLPATQRTSN---R-D-FS---- 317
MY        RPIVPPGRMGRPEPTSPTERNNLNKIS--G----HSRTLPSHSGRTS---SHD-PHMGHD 321
MG        NYNLHLGKFGKPEPYSPTERNNLNKPTVSV----HSRTLPNIGRSNR---DQE-IWMR-- 323
PC        HHVPPPGRLARPEPYSPTERSNLNKDEANSHQQHKANSTPSRTSVRGSTHQDDPSWSRGE 384
AC        PQAPPPGRLGRPEPYSPTERNNLNKMSDPS----RSSTLPAAHRTSG-YTRDDPQWGARG 387
LS        RHAPPPGRLGRPEPYSPTERNNLNKTSDPSSSSARSSTLPIAHRTSG-YPRDDSNWGARE 353
BG        RHAPPPGRLSRPEPYSPTERNNLNKPSEPSP--SRSSTLPSAHRTSG-YPHE--EWGSRE 350
                *::.:*** *****..*.*         :: : *

OB        ----------------RAVDPYVSHRS--RTFDPRINNR-----------------GRYF 342
MY        RSHDRSHDRSHERLHDRSHDRSHDHRYRTSSHSPDISRNSNN--------EQTKFLASHF 373
MG        ------------------EKSNIRHDLRSSSQSPDINKNSSD---------RTKILAGDF 356
PC        RN-P---DRGA-----S-----P---QGPSGRSPDLRNSER-----------SRILAHQF 416
AC        EG-GREHYEGG-----RDSRSLPRASPRPPSRSPDPGRGGAGGGGGGAGGNSAGRHDDRF 441
LS        L-----PGEGG-----RDSRSLPRPSPRASSRSPDPRGG--------------SRHDDRF 389
BG        R-----LNEGG-----RDSRSLPQPSVRSSSRSPDPRSG--------------GQHEDNF 386
                                          .*                         *

                                {start of C-terminal binding region}
OB        E----DPSLSV-------HMSDRRTETLPNGFKPKKR--KPENLDMCGDGTGGPVRHDSD 389
MY        D----EQASQNIGHEHGGRGRPRNASTLPNGFKPKGHKKKPEKLEMRSDSN-IPLRNDSD 428
MG        N----DPSSSS-------NRGSRPLKTVPNGFKPKGRKKKAEKMEMRSDSN-IPLNIDSD 404
PC        D-NAPAESSSTSGTSSEGRGRSRPSATLPNGFKPKGK--KTERYEMRSDIN-VPLKEDSD 472
AC        MAANQHEGSPARGGG--GAG-RARGVILPNGFKPKGR--KPEKYEMRTDSN-TALKEDSD 495
LS        MAASQQEGSPARGT-------RSRGGILPNGFKPKGR--KPEKYEMRSDSH-TALQEDSD 439
BG        MSASQAEGNS---P-------HSRGRIVPNGFKHRGR--KPEKYEMRSDSH-TALHEDSD 433
                 .                   :***** : :  * *. :*  *     :. ***

OB        -EDDDWC 395
MY        -DESDWC 434
MG        EDESDWC 411
PC        ED-DDWC 478
AC        EDDDDWC 502
LS        EDDDDWC 446
BG        EDDDDWC 440
           : .***
```

**Gastropod**	**AC**	**Aplysia californica**	**AVD53847.1**
**Gastropod**	**LS**	**Lymnaea stagnalis**	AAO83841.1
Gastropod	BG	Biomphalaria glabrata	KAI8771103.1
**Gastropod**	**PC**	**Pomacea canaliculata**	XP_025112132.1

Bivalve	MY	Mizuhopecten yessoensis	OWF37459.1
Bivalve	MG	Mytilus galloprovincialis	VDI13169.1
Cephalopod	Ob	Octopus bimaculoides	XP_052831401.1

Alignment of the channels are shown. Also, the start and end sites of the fragments used (Table 1) in determining binding of the SH3 domain of ApRBP are indicated as is the identified RBP binding site. Putative RBP sites and PDZ recognitions sites are in Red. *s and grey shading indicated completely conserved residues and represent sites of conserved function as determined by the ClustalW algorithm. Organisms and NCBI protein IDs are given.
